# Molecular Measurable Residual Disease Assessment before Hematopoietic Stem Cell Transplantation in Pediatric Acute Myeloid Leukemia Patients: A Retrospective Study by the I-BFM Study Group

**DOI:** 10.3390/biomedicines10071530

**Published:** 2022-06-28

**Authors:** Maddalena Benetton, Pietro Merli, Christiane Walter, Maria Hansen, Ambra Da Ros, Katia Polato, Claudia Tregnago, Jonas Abrahamsson, Luisa Strocchio, Edwin Sonneveld, Linda Fogelstrand, Nils Von Neuhoff, Dirk Reinhardt, Henrik Hasle, Martina Pigazzi, Franco Locatelli

**Affiliations:** 1 Department of Women’s and Children’s Health, Haematology-Oncology Clinic and Lab, University of Padova, 35128 Padova, Italy; maddalena.benetton@studenti.unipd.it (M.B.); ambra.daros@studenti.unipd.it (A.D.R.); katia.polato@unipd.it (K.P.); claudia.tregnago@unipd.it (C.T.); 2 Department of Pediatric Hematology and Oncology and Cell and Gene Therapy, IRCCS Ospedale Pediatrico Bambino Gesù, Sapienza University of Rome, 00165 Rome, Italy; pietro.merli@opbg.net (P.M.); luisa.strocchio@opbg.net (L.S.); 3 Department of Pediatric Hematology and Oncology, University Hospital Essen, 45147 Essen, Germany; christiane.walter2@uk-essen.de (C.W.); nils.vonneuhoff@uk-essen.de (N.V.N.); dirk.reinhardt@uk-essen.de (D.R.); 4 Department of Pediatrics, Aarhus University Hospital, 8000 Aarhus, Denmark; maria.hansen.05@regionh.dk (M.H.); hasle@dadlnet.dk (H.H.); 5 Department of Pediatrics, Institute of Clinical Sciences, “Sahlgrenska Academy” University of Gothenburg, 40010 Gothenburg, Sweden; vobjab@gmail.com; 6 Princess Máxima Center for Pediatric Oncology, 1013 GM Utrecht, The Netherlands; e.sonneveld-2@princesmaximacentrum.nl; 7 Department of Clinical Chemistry, Sahlgrenska University Hospital, 40010 Gothenburg, Sweden; linda.fogelstrand@clinchem.gu.se; 8 Department of Laboratory Medicine, Institute of Biomedicine, University of Gothenburg, 40010 Gothenburg, Sweden

**Keywords:** AML, HSCT, q-PCR, MRD, molecular genetics

## Abstract

Hematopoietic stem cell transplantation (HSCT) is a curative post-remission treatment in patients with acute myeloid leukemia (AML), but relapse after transplant is still a challenging event. In recent year, several studies have investigated the molecular minimal residual disease (qPCR-MRD) as a predictor of relapse, but the lack of standardized protocols, cut-offs, and timepoints, especially in the pediatric setting, has prevented its use in several settings, including before HSCT. Here, we propose the first collaborative retrospective I-BFM-AML study assessing qPCR-MRD values in pretransplant bone marrow samples of 112 patients with a diagnosis of AML harboring t(8;21)(q22; q22)*RUNX1::RUNX1T1*, or inv(16)(p13q22)*CBFB::MYH11*, or t(9;11)(p21;q23)*KMT2A::MLLT3,* or *FLT3*-ITD genetic markers. We calculated an ROC cut-off of 2.1 × 10^−4^ that revealed significantly increased OS (83.7% versus 57.1%) and EFS (80.2% versus 52.9%) for those patients with lower qPCR-MRD values. Then, we partitioned patients into three qPCR-MRD groups by combining two different thresholds, 2.1 × 10^−4^ and one lower cut-off of 1 × 10^−2^, and stratified patients into low-, intermediate-, and high-risk groups. We found that the 5-year OS (83.7%, 68.6%, and 39.2%, respectively) and relapse-free survival (89.2%, 73.9%, and 67.9%, respectively) were significantly different independent of the genetic lesion, conditioning regimen, donor, and stem cell source. These data support the PCR-based approach playing a clinical relevance in AML transplant management.

## 1. Introduction

Hematopoietic stem cell transplantation (HSCT) is the most effective option for preventing relapse in patients with acute myeloid leukemia (AML) achieving complete remission [1]. Prognosis of pediatric AML patients has improved over the last decade, with current long-term survival rates reaching 70% [2,3] and HSCT has contributed substantially to this improvement [2,4,5]. Nevertheless, relapse after HSCT remains the main cause of treatment failure [3,6]. Transplantation rates in ongoing trials with refined indications to HSCT vary from 8 to 29% and, notably, recent results have supported the use of HSCT in AML patients in first complete remission (CR) if they show features predicting high risk of recurrence [7]. Achievement of morphological CR, defined as the presence of normal hematopoiesis and ≤5% blasts in the bone marrow, is important to optimize the outcome of patients given HSCT. However, to date, no studies in pediatric AML have analyzed the impact of molecular minimal residual disease (MRD) measurement before HSCT on transplant outcome. Moreover, given the availability of different methods to evaluate MRD (with different sensitivity), distinguishing diverse subgroups with different risks of post-transplant relapse could help implement novel intervention strategies aimed at reducing treatment failure. Measurable residual disease determined by quantitative polymerase chain reaction (qPCR-MRD) has increased the ability to monitor therapy response in AML [8,9,10]; in clinical trials conducted in adults, there is robust evidence that measurable residual disease determined by quantitative polymerase chain reaction (qPCR-MRD) increased the ability to monitor therapy response and improved prognostic precision [8,9,10] with respect to other techniques, including flow cytometry, leading to a refined risk stratification [11,12]. Indeed, recently, it has been demonstrated in *CBF*-rearranged and *NPM1*-mutated patients that qPCR-MRD played an independent prognostic role when assessed at different timepoints, including before transplant [13]. However, despite initial promising results for certain genetic lesions [14], the role of qPCR-MRD monitoring in childhood AML to define a deeper remission status is still controversial, and currently defined by flow cytometry [15,16,17]. By the way, there is a proportion of patients with MRD negativity experiencing relapse which can be due to (i) the acquisition of new disease markers/immunophenotype shift or (ii) low sensitivity and specificity of the method used (e.g., limit of manual gating strategies used to analyze multidimensional data) [15]. Over the last two decades, great advances in deciphering the genetic landscape of pediatric AML have been achieved [18,19], and therefofe, a suitable qPCR-MRD biomarker related to recurrent chromosomal rearrangements and gene mutations is now available in up to 75% of patients [19,20]. The role of MRD measurement before HSCT has been established for several hematological malignancies and in adult AML [21,22,23,24], but, so far, no data have been reported in pediatric AML. Furthermore, the concept of “molecular remission”, introduced for the first time in 2003 by the International Working Group guidelines to refine treatment response adults in AML [20], has never reached a consensus within large cooperative pediatric groups.

Here, we describe the results of an international retrospective BFM collaborative retrospective study in a large cohort of AML pediatric patients on qPCR-MRD monitoring and its prognostic role before HSCT. This analysis was possible due to harmonization of methods, and reports qPCR-MRD analyses by different MRD thresholds, finding that disease levels are associated with different probabilities of overall survival, relapse and transplant-related mortality, to be further considered in multidisciplinary discussion before transplant.

## 2. Materials and Methods

### 2.1. Patients and Samples

Included in the study are patients (i) with de novo AML (*n* = 112) other than acute promyelocytic leukemia; (ii) aged 0–18 years; (iii) diagnosed between 2002 and 2016 in one of the three consortia participating in this collaborative study; (iv) harboring isolated molecular lesion among t(8;21)*RUNX1::RUNX1T1*, inv(16)*CBFB::MYH11*, t(9;11)*KMT2A::MLLT3,* or *FLT3*-ITD. The AIEOP (Associazione Italiana Ematologia Oncologia Pediatrica) included *n* = 63 patients, Berlin–Frankfurt–Münster (BFM) study group included *n* = 11 patients, and the NOPHO (Nordic Society of Paediatric Haematology and Oncology) included *n* = 38 patients. The approval for this study was obtained from the institutional review board of each participating center. All patients provided written informed consent within each trial in accordance with the Declaration of Helsinki, obtained from patients’ parents or legal guardians. French-American-British (FAB) morphological classification, immune-phenotype analysis, and molecular characterization were performed at the laboratory of Pediatric Hematology of the University Hospital in Padova for the AIEOP cases and patients were enrolled in the AIEOP-AML2002/01 trial. For the BFM-SG group, samples were provided by the Clinic for Pediatrics III of the University Hospital of Essen (and patients were treated according to AML-BFM 2012 trial). As regards NOPHO patients, samples were collected at the Department of Clinical Chemistry at the Sahlgrenska University Hospital in Gothenburg or at the Department of Hematology Hemodiagnostic Laboratory of the Aarhus University Hospital (and patients were included in NOPHO AML-2004 or NOPHO-DBH AML-2012 trials). Post-remission treatment was determined according to genetic and cytogenetic criteria, reserving HSCT in first or second CR (CR1, or after a morphological relapse when in second remission phase, CR2). For this study, MRD results were included if the sample was taken within 4 weeks before HSCT and patients did not receive any further therapy between sampling and the start of conditioning.

### 2.2. RNA Extraction and Retro-Transcription

Mononuclear cells from bone marrow (BM) samples were isolated by density gradient using Lymphoprep™ (Stemcell Technologies, Vancouver, BC, Canada). RNA extraction and reverse transcription were performed by groups, as previously reported [25].

### 2.3. Molecular Marker Identification by Polymerase Chain Reaction

All patients were screened at diagnosis for t(8;21)*RUNX1::RUNX1T1*, inv(16)*CBFB::MYH11*, t(9;11)*KMT2A::MLLT3*, and *FLT3*-ITD by reverse transcription polymerase chain reaction (RT-PCR) with specific primers previously validated to detect the lesions [26,27,28,29]. Shifted primers were used to confirm the positivity of the rearrangement/mutation and to define the breakpoint for each case. All AML cases underwent karyotyping or FISH at diagnosis to confirm the lesion and characterize blasts’ karyotypes.

### 2.4. qPCR-MRD Detection

For each rearrangement included in the study (namely t(8;21) *RUNX1::RUNX1T1,* inv(16)*CBFB::MYH11*, t(9;11)*KMT2A::MLLT3*, and *FLT3*-ITD), we set up a real-time quantitative reverse transcription polymerase chain reaction (qPCR) to quantify AML-specific transcripts and monitor qPCR-MRD, as previously described [25,26,27,28,29]. The local laboratories tested the performance of all assays by evaluating each new batche of primer/probe sets by standard curve dilutions. Efficiency was in a dynamic range of at least 4 logs, with 6 logs as the maximum sensitivity reached.

### 2.5. Quality Control of Standard Operating Procedures (SOPs)

Patients’ samples were exchanged among laboratories to compare SOP performances. In particular, samples of one patient at diagnosis and follow-up for each genetic lesion from each reference laboratory were sent to the other laboratories. Samples were used to perform cDNA preparation locally following each lab’s SOPs, as well as qPCR. Quantification cycle (Cq) values together with calculated ∆Cq and qPCR-MRD values were disclosed and compared among laboratories to validate the previously discussed and harmonized methods.

### 2.6. Statistical Methods

Significance among groups was analyzed using the χ2 test and Fischer’s exact test. Overall survival (OS) was calculated from the date of the HSCT to the time of death due to any cause or time of last contact. Event-free survival (EFS) was calculated from the date of the HSCT to the last follow-up or first event (failure to engraft, relapse, death in remission, secondary malignancies, whichever occurs first). Relapse-free survival (RFS) was calculated from the date of the HSCT to the last follow-up or relapse event. OS, EFS, and RFS were estimated using the Kaplan–Meier method and groups were compared by using the log-rank test. Treatment-related mortality (TRM) and cumulative incidence of relapse (CIR) were calculated as cumulative incidence curves to adjust the estimates for the appropriate competing risks. Gray’s test was used to assess, in univariate analyses, differences between cumulative incidences using the “cmprsk” package for R software. A multivariable analysis was performed with Cox regression analysis considering the ages of patients at HSCT, type of donor, CR status, and qPCR-MRD reduction. Graphs and associated statistical analyses were generated using GraphPad Prism 8. A *p*-value <0.05 was considered to be statistically significant.

## 3. Results

### 3.1. Inter-Laboratory Quality Control (QC)

This study was retrospective in nature, and all study groups had developed their methodology independently. Therefore, we formed a network of reference laboratories within iBFM, EuMolNet, to share methods and expertise. To verify uniformity among the laboratories, we performed four different QC rounds that consisted of exchanging RNA among labs and performing the qPCR-MRD measurements of different molecular aberrations. The QC results showed that inter-laboratory testing of collected patients’ samples were not statistically different and qPCR-MRD values remained in the same log reduction in each lab that performed the analysis (Figure S1). These findings guaranteed that the results obtained were robust and suitable for the collaborative qPCR-MRD study presented here.

### 3.2. Survival According to the Type of Remission

Given the retrospective nature of this study, we enrolled patients for whom genetic screening was available at diagnosis and material biobanked before transplant. Therefore, AML harboring t(8;21), inv(16), t(9;11), and FLT3-ITD lesions were available among all institutions. Clinical and molecular details of the patients enrolled in the studies are shown in Table 1 (Figure S2).

**Figure 1:**
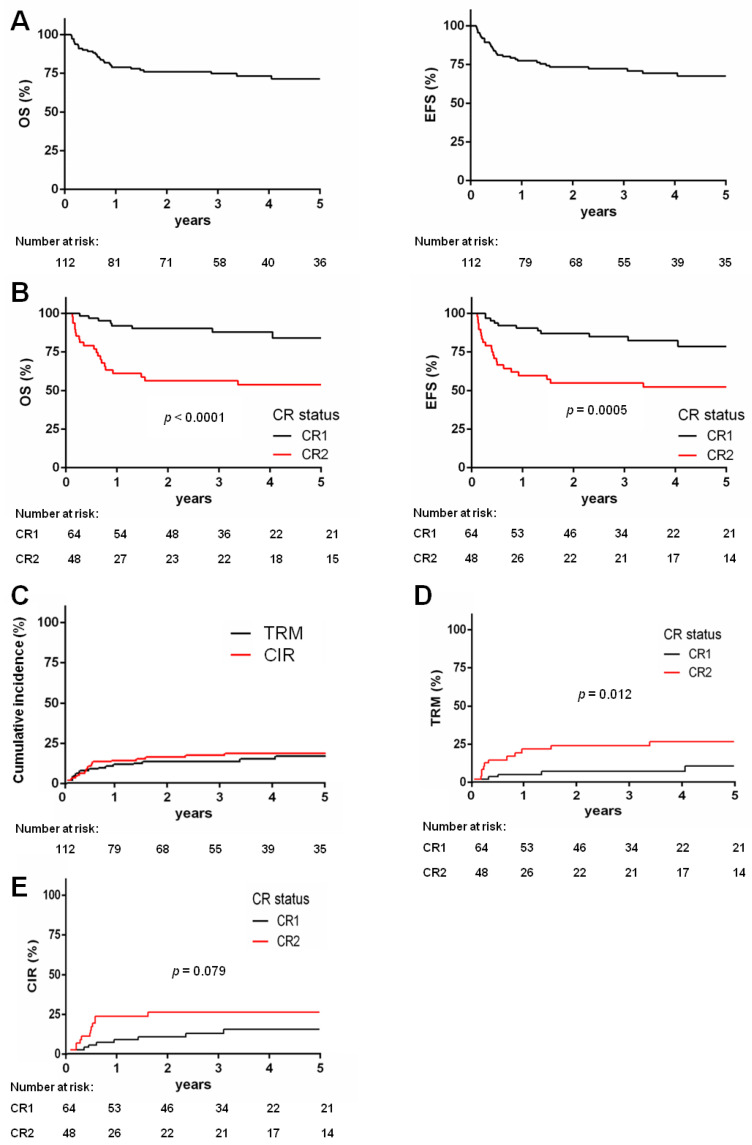
Survival of the whole patients’ cohort according to the remission status before HSCT: (**A**) Kaplan–Meier estimates showing 5-year probability of overall survival (OS, left, survival 71.4%) and event-free survival (EFS, right, survival 67.6%) for the whole cohort of patients enrolled (*n* = 112); (**B**) five-year probability of OS (left) for children receiving HSCT in CR1 (*n* = 64) or CR2 (*n* = 48) (84% vs. 53.9%, *p* < 0.0001) and EFS for patients transplanted in CR1 or CR2 (78.6% vs. 52.3%, *p* = 0.0005); (**C**) cumulative incidence of treatment-related mortality (TRM) in the whole population of children given HSCT (black line, *n* = 112, 13.5%), and cumulative incidence of relapse (CIR) in the whole population of AML patients given transplantation (red line, *n* = 112, 17.1%); (**D**) cumulative incidence of transplant-related mortality in patients who underwent transplantation in CR1 (*n* = 64) or CR2 (*n* = 48) (8.8% vs. 24.4%, *p* = 0.012); (**E**) cumulative incidence of relapse in patients who underwent transplantation in CR1 (*n* = 64) or CR2 (*n* = 48) (12.7% vs. 23.3%, *p* = 0.079).

Sixty-four out of 112 patients were transplanted in CR1 and 48 in CR2. With a median follow-up after transplant of 3.1 years (range 0.1–16.3 years); 29 patients (26%) died, 15 cases because of transplant-related causes and the remaining 14 cases because of leukemia recurrence. The five-year OS of the entire cohort was 71.4% (95% CI 61.1–79.4), while the 5-year EFS was 67.6% (95% CI 57.1–76) (Figure 1A). The 64 patients transplanted in CR1 showed a 5-year OS of 84% (95% CI 68.9–92.2), while the 48 children who receive HSCT in CR2 had an OS of 53.9% (95% CI 38.3–67.2, *p* < 0.0001). Similarly, the 5-year EFS was 78.6% (95% CI 63.4–88) for patients given HSCT in CR1 and 52.3% (95% CI 36.9–65.6) for CR2 patients (*p* = 0.0005, Figure 1B). TRM was 13.5% and CIR was 17.1% for the whole cohort (Figure 1C); TRM significantly increased to 24.4% (95% CI 12.9–37.9) for patients transplanted in CR2 with respect to a value of 8.8% observed in patients given HSCT in CR1 (95% CI 2.4–20.4, *p* = 0.0012, Figure 1D). CIR was 12.7% (95% CI 5.5–23.3) and 23.3% (95% CI 12.3–36.2) for CR1 and CR2 patients, respectively (*p* = 0.079, Figure 1E). The source of stem cells did not have impact on outcome, whereas the type of donor did; indeed, patients transplanted from a matched-related donor had a better OS as compared with those transplanted from a matched unrelated donor, as observed in those few ones who received an autologous transplant (*n* = 8) (*p* = 0.034, Figure S3A,B). Moreover, there was no influence of the type of conditioning regimen used (all enrolled patients received a myeloablative preparation) (Figure S3C). When considering age, we did not find significant impact on outcomes (Figure S4).

### 3.3. qPCR-MRD Threshold 2.1 × 10^−4^ Is Associated with HSCT Outcome

**Figure 2:**
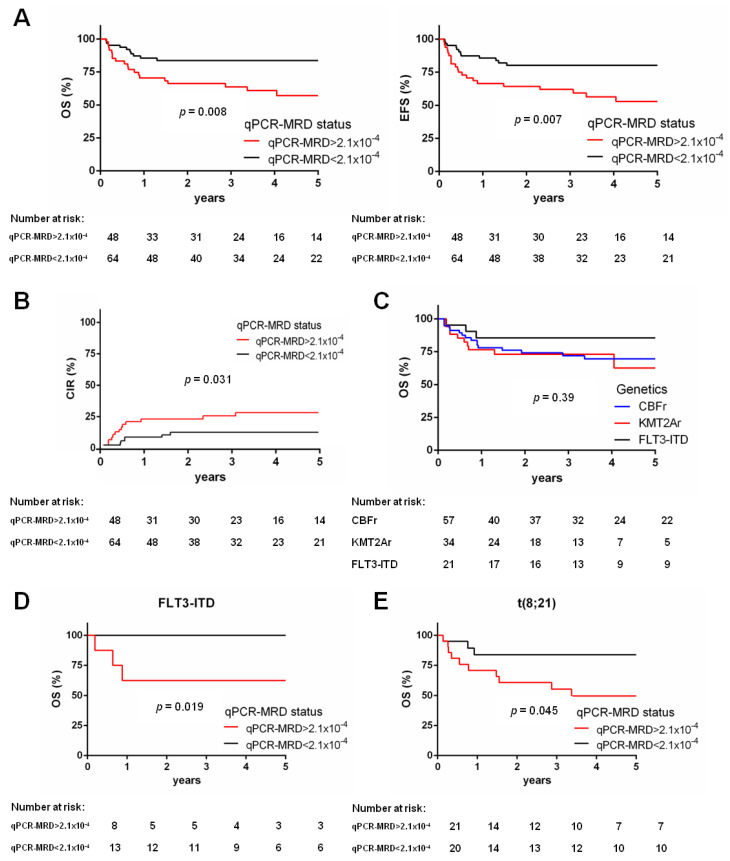
qPCR-MRD values above 2.1 *×* 10^−4^ impact on transplantation outcome: (**A**) Kaplan–Meier curves showing that patients who underwent HSCT with qPCR-MRD values above 2.1 *×* 10^−4^ (*n* = 48) have a worse OS with respect to patients receiving transplant with qPCR-MRD values below 2.1 *×* 10^−4^ (*n* = 64) (57.1% vs. 83.7%, *p* = 0.008), EFS for the same groups of patients (52.9% vs. 80.2%, *p* = 0.007); (**B**) cumulative incidence of relapse for cases with qPCR-MRD values above 2.1 *×* 10^−4^ (*n* = 48) and patients with qPCR-MRD values below 2.1 *×* 10^−4^ (*n* = 64) (26% vs. 10%, *p* = 0.031); (**C**) five-year OS for patients according to their genetics: *CBF*r (*n* = 57), *KMT2A*r (*n* = 34), and *FLT3*-ITD (*n* = 21) (survival 69.6% vs. 62.7% vs. 85.5%, respectively, *p* = 0.39); (**D**) Kaplan_Meier survival curves of OS for children with *FLT3*-ITD mutation given transplantation with qPCR-MRD values above 2.1 *×* 10^−4^ (*n* = 8) or with qPCR-MRD values below 2.1 *×* 10^−4^ (*n* = 13) (62.5% vs. 100%, *p* = 0.019); (**E**) five-year probability of OS for children with t(8;21)*RUNX1::RUNX1T1* rearrangement given HSCT with qPCR-MRD values above 2.1 *×* 10^−4^ (*n* = 21) or with qPCR-MRD values below 2.1 *×* 10^−4^ (*n* = 20) (49.7% vs. 83.8%, *p* = 0.045).

Evaluable pre-HSCT BM samples collected within 4 weeks before HSCT were measured for residual disease by qPCR. Given the lack of reported qPCR-MRD threshold associated with HSCT outcomes, we performed an ROC analysis, finding that qPCR-MRD values below 2.1 *×* 10^−^^4^ (or undetectable associated to values below the limit of detection) for 64 patients (57%) was associated with an improved outcome. Indeed, those 64 patients with qPCR-MRD values below 2.1 *×* 10^−^^4^ had a 5-year OS of 83.7% (95% CI 71.7–90.9), whereas those with qPCR-MRD values above the cut-off had an OS of 57.1% (95% CI 40.6–70.6, *p* = 0.008, Figure 2A) Similar significant results were obtained for EFS in these two groups (*p* = 0.007, Figure 2A). Eighteen (16%) patients relapsed at a median time of 5.2 months after transplantation (75% within the first year). Among them, children who had qPCR-MRD values above 2.1 *×* 10^−^^4^ had a higher risk to relapse (26%) as compared with those with lower qPCR-MRD values (qPCR-MRD < 2.1 *×* 10^−^^4^, 10%, *p* = 0.031, Figure 2B). Of note, the type of genetic lesion, namely *CBF*r, *KMT2A*r, and *FLT3*-ITD, did not correlate with outcome (*p* = ns, Figure 2C and Figure S5A). Analyzing the influence of qPCR-MRD values on outcome for each single rearrangement, we found that qPCR-MRD values below 2.1 *×* 10^−^^4^ significantly influenced OS and EFS in *FLT3*-ITD patients (OS 100% versus 62.5%, *p* = 0.019, Figure 2D, EFS in Figure S5B), and in t(8;21)*RUNX1::RUNX1T1*-rearranged patients (OS 83.8% versus 49.7%, *p* = 0.045, Figure 2E, EFS in Figure S5C). On the contrary, for the remaining genetic lesions, the qPCR-MRD value did not play a significant role (Figure S5D–F), however, the power of these analyses is limited by a reduced sample size.

### 3.4. qPCR-MRD Threshold 1 × 10^−2^ Refines HSCT Outcome Prediction

**Figure 3:**
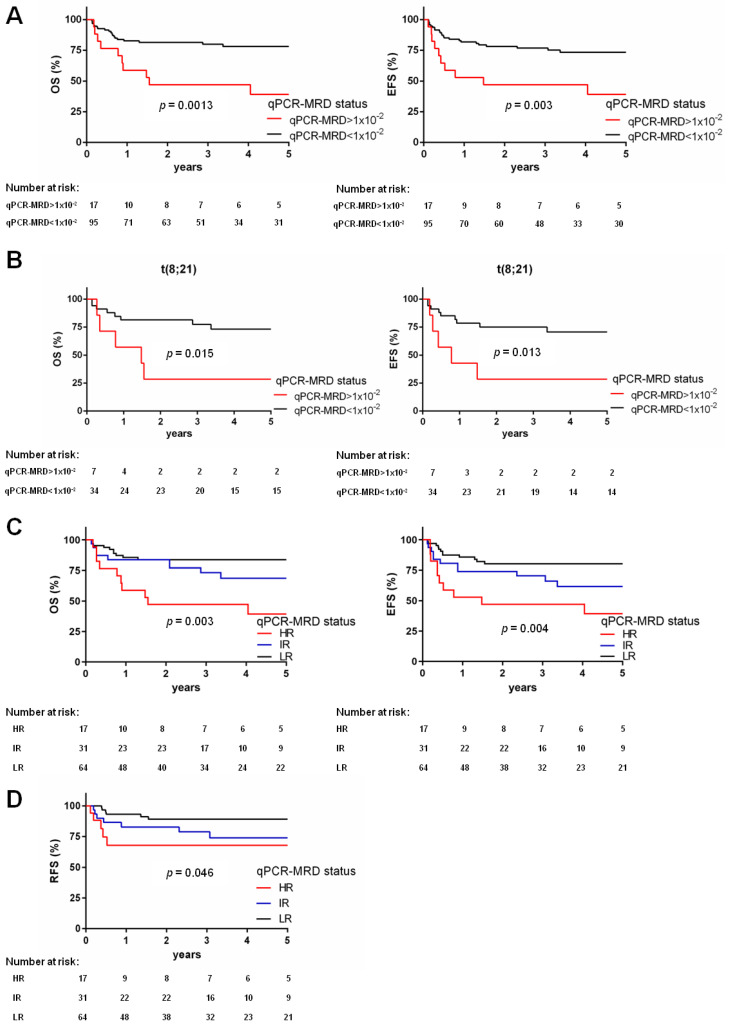
Two qPCR-MRD thresholds generate a risk stratification model predicting the survival of patients: (**A**) Survival estimates for patients receiving HSCT with qPCR-MRD values above 1 *×* 10^−2^ (*n* = 17) and qPCR-MRD values below 1 *×* 10^−2^ (*n* = 95). Curves show OS (left, 39.2% vs. 78.2%, *p* = 0.0013) and EFS (right, 39.2% vs. 73.4%, *p* = 0.003); (**B**) five-year survival curves for patients harboring t(8;21)*RUNX1::RUNX1T1* rearrangement given HSCT with qPCR-MRD values above 1 *×* 10^−2^ (*n* = 7) and qPCR-MRD values below 1 *×* 10^−2^ (*n* = 34): OS (28.6% vs. 73.2%, *p* = 0.015) and EFS (28.6% vs. 70.7%, *p* = 0.013); (**C**) Kaplan–Meier curves of OS or patients who underwent HSCT with qPCR-MRD values above 1 *×* 10^−2^ (HR, *n* = 17), 2.1 *×* 10^−4^ < qPCR-MRD values < 1 *×* 10^−2^ (IR, *n* = 31), and qPCR-MRD values below 2.1 *×* 10^−4^ (LR, *n* = 64) (survival 39.2% vs. 68.6% vs. 83.7%, respectively, *p* = 0.003) and EFS (survival 39.2% vs. 61.6% vs. 80.2%, *p* = 0.004); (**D**) relapse-free survival of patients according to three risk stratification model: qPCR-MRD values above 1 *×* 10^−2^ (HR, *n* = 17), 2.1 *×* 10^–4^ < qPCR-MRD values < 1 *×* 10^−2^ (IR, *n* = 31), and qPCR-MRD values below 2.1 *×* 10^−4^ (LR, *n* = 64) (67.9% vs. 73.9% vs. 89.2%, respectively, *p* = 0.046).

Among the patients who underwent HSCT with high detectable qPCR-MRD values (*n* = 48), we observed very heterogeneous qPCR-MRD values. Thus, we stratified patients at a higher qPCR-MRD threshold, namely qPCR-MRD values above 1 *×* 10^−^^2^. This value identified a cohort of 17 very high-risk patients (HR) with worse OS and EFS (OS 39.2%, versus 78.2% for the other 95 patients, *p* = 0.0013, and EFS 39.2% versus 73.4%, *p* = 0.003, Figure 3A). This cut-off clearly defined that these patients (15% of the total cohort) underwent HSCT with a low qPCR-MRD reduction, resulting in a dismal outcome. These patients were equally distributed in CR1 (*n* = 8) or CR2 (*n* = 9); they were heterogeneous for genetic markers (t(8;21) *n* = 7, inv(16) *n* = 2, t(9;11) *n* = 5, and FLT3-ITD *n* = 3), and for the type of HSCT received (MUD *n* = 8, matched family donor *n* = 9) (Table S1). Analyzing outcomes according to the 1 *×* 10^−^^2^ qPCR-MRD reduction threshold in each genetic class, we found that among the HR patients for qPCR-MRD before HSCT, those patients harboring t(8;21)*RUNX1::RUNX1T1* had the worst prognosis (OS 28.6% for qPCR-MRD values above 1 *×* 10^−^^2^ versus 73.2% for qPCR-MRD values below 1 *×* 10^−^^2^, *p* = 0.015; and EFS 28.6% versus 70.7%, respectively, *p* = 0.013, Figure 3B); notably, most of these cases received HSCT while in CR2 (six out of seven cases). Regarding *FLT3*-ITD, t(9;11)*KMT2A::MLLT3*, and inv(16)*CBFB::MYH11* only three, five, and two cases, respectively, were included in the HR class (Table S1) because of a very good blast clearance and transplantation in CR1 for most of these patients (Figures S2 and S6).

### 3.5. Model of Pre-HSCT Risk Stratification by qPCR-MRD

With the purpose to better predict outcome after HSCT, we analyzed the influence of these two different qPCR-MRD thresholds on outcome, proposed a model where the two significant cut-offs generated three risk groups, namely low (LR), intermediate (IR), or high (HR). This stratification defined patients with qPCR-MRD negative or <2.1 *×* 10^−^^4^ values as belonging to the LR group (*n* = 64, 57% of the whole cohort); in this group, 6 out of 64 (9%) patients experienced relapse and ten patients died. Seventeen patients with qPCR-MRD values above 1 *×* 10^−^^2^ (15%) were allocated to the HR group, 5/17 patients relapsed (29%) experiencing a poor outcome (EFS 39.2%) (Table S2). Finally, in the IR group (*n* = 31, 28%), the qPCR-MRD values were in between the two cut-offs; 7 out of these 31 patients (22%) had a relapse. In detail, the estimated 5-year OS of the three risk groups was 83.7% for LR, 68.6% for IR, and 39.2% for HR, while the 5-year EFS was 80.2%, 61.6%, and 39.2% (*p* = 0.003 and *p* = 0.004, respectively, Figure 3C). Notably, this model confirmed a significantly reduced relapse-free survival for those patients with higher qPCR-MRD values (HR, RFS = 67.9%; IR, RFS = 73.9%; LR, RFS = 89.2%, *p* = 0.046, Figure 3D and Figure S7). Finally, by multivariate Cox regression analysis, our proposed qPCR-MRD stratification model was found to be an independent factor (hazard ratio 0.47, *p* = 0.0011) together with disease status at time of allograft (hazard ratio 4.5 for CR2, *p* = 0.0006) influencing OS and EFS (Table S3).

## 4. Discussion

Advances in genetic profiling have, in part, clarified the complex dynamics of AML. Moreover, a large number of molecular markers that not only influence risk stratification at diagnosis, but also may help in monitoring response to therapy are now available [18]. In this regard, protocols used by different cooperative groups differ in genetic marker selection [30,31], risk stratification, MRD monitoring, and indications to HSCT [24,32]. In this study, we managed some issues related to qPCR-MRD, trying to assess whether it could improve risk stratification to ultimately personalize post-transplant strategies. We assessed and established optimal qPCR assays validated independently by five reference laboratories being included in the BFM-pediatric AML study group (Italy, Germany, Sweden, the Netherlands, and Denmark), following the adult AML landmark experience in this methodology implementation, based on the Europe Against Cancer (EAC) program and the European Leukemia Network (ELN) [31,33,34,35]. We defined a common interpretation of qPCR-MRD, resulting into high sensitivity and high specificity to detect blasts and determining MRD values, bypassing the more heterogeneous results obtained by multiparameter flow cytometry analysis (MFC) due to site-specific differences in MFC methodology and operators, antigens, and fluorochromes used, methods for cell lysis, number of events collected, and AML immunophenotype shift [36]. This collaborative international study retrospectically collected qPCR-MRD values of 112 AML cases harboring a recurrent molecular marker, being monitored before transplant received in CR1 for high-risk features or in CR2 after a disease recurrence, accordingly to each different national trial stratification. The associations between qPCR-MRD and post-HSCT relapse and outcome were robust, and seen within all cases, regardless of the genetic risk and trial the patients were enrolled in. However, given the nature of this study of qPCR-MRD validation, we were unable to account for inter-study differences in the selection of patients for HSCT, different post-remission treatments, heterogeneity in transplant conditioning regimens, graft characteristics, and immunosuppression.

We found that patients with undetectable qPCR-MRD had a superior long-term survival, independent from the state of CR1 or CR2 remission at HSCT, the type of conditioning regimen, donor employed, and genetic rearrangement and that the use of HSCT in CR2 was independently associated with a worse outcome in this subgroup of AML patients [37,38,39,40,41,42,43]. We documented that detectable higher qPCR-MRD values had an overall adverse effect on transplant outcome. In particular, we determined that the most informative cut-off (2.1 × 10^−4^) was able to discriminate patients with a low risk to relapse (10.4%) and an extremely good OS (82.8%). We also explored a higher cut-off equivalent to 1 × 10^−2^ of qPCR-MRD that allowed the identification of a group of 17 patients with high qPCR-MRD values (>1 × 10^−2^) and very poor OS (i.e., below 40%). Thanks to this novel partitioning of AML cases according to qPCR-MRD values, we proposed a pre-HSCT allocation to low-, intermediate-, and high-risk groups. This stratification, supported by relapse free survival, could help personalizing transplant management in order to improve clinical outcome. Of interest, the 17 patients defined as HR cases could be recognized exclusively by the qPCR-MRD values. Notably, analyzing subgroup of patients according to their genetic lesion, we showed that qPCR-MRD reduction in t(8;21)-rearranged (*n* = 41) and *FLT3*-ITD (*n* = 21) patients significantly predicted patients outcome, as previously found after induction course [14,29,44]. For the *KMT2A*-rearranged AML cases (*n* = 34), we did not find a significant correlation with qPCR-MRD values; however, most of these patients had low qPCR-MRD values at the time of HSCT. This observation of a good qPCR-MRD reduction by both the *FLT3*-ITD and t(9;11)*KMT2A::MLLT3* subgroups, together with the fact that, in this setting, patients were mainly transplanted in CR1, fosters the relevance of CR1 and qPCR-MRD in planning an HSCT with high rate of success in both subgroups [7]. Management of patients with low qPCR-MRD reduction remains controversial, and a key question is whether the type of conditioning can affect residual disease, and thereby improve outcome. Studies mostly in adult AML patients have provided conflicting results due to specific transplant conditioning [45,46]. A recent report by Hourigan and colleagues on adult AML patients in CR randomized to receive either myeloablative or reduced-intensity conditioning (RIC) documented that patients with measurable MRD had an increased risk of relapse if treated with RIC [47]. Here, all pediatric patients received a myeloablative treatment, and we observed no effect on survival according to different types of conditioning.

Our study establishes the importance of pre-HSCT qPCR-MRD, attempting a qPCR-MRD based risk-stratification to identify the majority of patients experiencing adverse events after HSCT, and supports that they might be discussed for alternative approaches either before (such as the use of refined conditioning regimens, immunotherapy, or targeted therapy to reduce the qPCR-MRD value) [48] or after HSCT (such as the rapid tapering of immunosuppression, the use of donor-lymphocyte infusions, or specific agents such as tyrosine-kinase inhibitors).

Finally, this study supports that MRD detection has strong clinical relevance during the whole AML course and that technological advances in the molecular field allowed to increase sensitivity and specificity, making it an essential tool with the ability to complement morphology and immunophenotypic analysis, particularly for compelling clinical decisions. However, since previous studies have shown a high concordance between qPCR and MFC residual disease values, we support that the prognostic role of MRD is not influenced by the detection method [15,29], thus, we consider the MRD risk model described here to be a further tool to be validated in larger cohorts.

## 5. Conclusions

This international retrospective I-BFM-AML group study, regardless of its limitations, strengthens the need for collaborative networks and the comparison with other techniques supports the role of pediatric residual disease monitoring before HSCT to refine therapeutic decisions. Furthermore, new challenges in molecular approaches to improve qPCR are being faced by using next-generation sequencing [49,50], where multiplex quantification of mutations [51] could improve the ability to track molecular AML residual disease, as well as cover all patients lacking, for now, a qPCR marker.

## Figures and Tables

**Table 1:** Patients’ characteristics.

	AIEOP	NOPHO	BFM	Total
**n° pts**	63	38	11	112
**Age (average)**	7.9	9.1	9.8	8.9
**Gender**				
male	37	19	7	63
female	26	19	4	49
**WBC (average) (*n* = 107)**	50,382	59,245	52,635	53,347
**Genetics**				
**Standard risk**				
t(8;21)*RUNX1::RUNX1T1*	20	17	4	41
inv(16)*CBFB::MYH11*	8	6	2	16
**High risk**				
t(9;11)*KMT2A::MLLT3*	19	10	5	34
*FLT3*-ITD	16	5	0	21
**Karyotype (*n* = 82)**				
complex	6	8	1	15
**Type of remission**				
CR1	53	7	4	64
CR2	10	31	7	48
**pts status**				
relapse post HSCT (n°)	7	11	0	18
death (n°)	8	18	3	29
**Median Follow-up (years)**	3.3	2.3	1.4	3.1
range	0.3–14	0.1–16	0.1–4	0.1–16
**Type of HSCT (*n* = 108)**				
MUD-ALLO	21	28	4	53
SIBLING-RELATED	34	10	3	47
AUTOLOGOUS	8	0	0	8
**Source (*n* = 94)**				
BM	45	12	4	61
PB	15	14	1	30
CB	3	0	0	3
**Preparative regimen before HSCT**				
BUS-based	52	11	2	65
TBI-based	6	0	0	6
other	5	15	5	25

## Data Availability

Data sharing is not applicable to this article as no datasets were generated or analyzed during the current study.

## Supplementary Materials

The following supporting information can be downloaded at: https://www.mdpi.com/article/10.3390/biomedicines10071530/s1. Figure S1: Inter-laboratory quality control (QC); Figure S2: Schematic diagram showing the number of patients in each part of the study; Figure S3: Effect of transplant-related variables on survival; Figure S4: Impact of patients’ age on clinical outcome; Figure S5: Genetics and qPCR-MRD status role in HSCT; Figure S6: Bar representation of the percentage of patients belonging to LR, IR and HR groups according to their genetic lesions; Figure S7: Relapse-free survival from HSCT according to qPCR-MRD values; Table S1: Clinical and biological characteristics of high risk patients for qPCR-MRD reduction before HSCT; Table S2: Clinical, molecular, and transplant variables in each qPCR-MRD-defined group; Table S3: Multivariate analyses.
