# Supplementary material for: Molecular Measurable Residual Disease Assessment before Hematopoietic Stem Cell Transplantation in Pediatric Acute Myeloid Leukemia Patients: A Retrospective Study by the I-BFM Study Group
Source: Biomedicines. 2022 Jun 28;10(7):1530. doi: 10.3390/biomedicines10071530 (PMC9313005; doi:10.3390/biomedicines10071530)
Supplement: Supplementary file 1 [file biomedicines-10-01530-s001.zip › biomedicines-1757946-supplementary.pdf]

Supplemental Material

Molecular measurable residual disease assessment before hematopoietic stem cell transplantation in pediatric acute myeloid leukemia patients: a retrospective study by the I-BFM Study Group.

Benetton M. and Merli P. *et al.*

SUPPLEMENTAL METHODS

qPCR-MRD evaluation

For each rearrangement included in the study (t(8;21)*RUNX1::RUNX1T1*, inv(16)*CBFB::MYH11*, t(9;11)*KMT2A::MLLT3,* and *FLT3*-ITD), we set up a real-time quantitative reverse transcription polymerase chain reaction (RT-qPCR) to quantify tumor-specific transcripts and monitor qPCR-MRD, taking advantage of Taqman technology on an ABI 7900HD platform (Applied Biosystems).
Each laboratory tested the performance of all assays by evaluating each new batche of primer/probe sets by standard curve dilutions for specificity and sensitivity. Briefly, a serial dilution curve ranging from 1 to 10^{-6} of the positive control was performed, and RT-qPCR was performed. An acceptable sensitivity was obtained when at least the 10^{-4} was reached with a detectable Cq value ≤40. Specificity was considered to be optimal (100%) when the control cDNA (negative for the rearrangement under evaluation) was undetectable (tested in triplicate). Efficiency between 90 and 110% and a dynamic range of at least 4 logs must be reached, with 6 logs as maximum sensitivity reached. All samples were tested in triplicate; the Cq triplicate values should preferably not differ more than 1 Cq and were expressed as a ratio between the leukemia-specific transcript and the housekeeping genes to adjust for variations in mRNA quality or efficiencies in cDNA synthesis, and then measured relative to tumor-specific transcript levels at diagnosis by using the ΔCt method calculation. A Cq value >28 is considered unsuitable for the housekeeping gene.

SUPPLEMENTAL TABLES

Table S1: Clinical and biological characteristics of high risk patients for qPCR-MRD reduction before HSCT.

	**Number**	**Percentage**
**Patients**	17	
	17/112	15.20%
**Gender (M/F)**	7/10	41.2%/58.8%
**Median WBC at diagnosis (*n* = 15)**	42900	
**Karyotype (normal/aberrant) (*n* = 14)**	7/7	
**Median age at diagnosis (years)**	9.7	
**Median age at HSCT (years)**	11.3	
**HSCT type**		
Allogenic	17	100.00%
related	9	52.90%
MUD	8	47.10%
**CR1/CR2**	9/8	52.9%/47.1%
**HSCs source (*n* = 14)**		
BM	11	64.70%
PB	3	17.60%
**Median follow-up (months)**	47.2	
**Status (alive/dead)**	7/10	41.2%/58.8%
**Conditioning regimen (*n* = 14)**		
BUS-based	10	58.80%
TBI	0	0%
other	4	23.50%
**Molecular Marker:**		
t(8;21)*RUNX1::RUNX1T1*	7	41.2%
inv(16)*MYH11::CBFB*	2	11.8%
KMT2A-rearranged	5	29.4%
FLT3-ITD	3	17.60%

Table S2: Clinical, molecular, and transplant variables in each qPCR-MRD-defined group.

***n* = 112**	**High (*n* = 17)**	**Intermediate (*n* = 31)**	**Low (*n* = 64)**
**Median age at diagnosis**	9.7	12.5	8.1
**Gender (M/F)**	7/10	18/13	38/26
**CR1/CR2**	9/8	17/14	38/26
**Deaths**	10 (59%)	9 (29%)	10 (16%)
**Relapses**	5 (29%)	7 (22.6%)	6 (9%)

Table S3: Multivariate analyses.

OS				
	**Hazard ratio**	**Lower 95% CI**	**Upper 95% CI**	**p-Value**
**SIB_MUD_OTHER**	1.0380	0.5876	1.8340	0.8973
**GENDER**	0.6009	0.2857	1.2640	0.1793
**qPCR-MRD (3 groups)**	0.4701	0.2985	0.7404	0.0011
**Date of HSCT** (2001-2010 vs 2011-2018)	0.6860	0.2910	1.6170	0.3890
**CR1/CR2**	4.4940	1.9100	10.5700	0.0006
***FLT3*-ITD/*CBF*r/*KMT2A*r**	1.7280	0.8745	3.4130	0.1155

EFS				
	**Hazard ratio**	**Lower 95% CI**	**Upper 95% CI**	**p-Value**
**SIB_MUD_OTHER**	1.4110	0.8474	2.3490	0.1857
**GENDER**	0.5030	0.2481	1.0200	0.0567
**qPCR-MRD (3 groups)**	0.4810	0.3123	0.7406	0.0009
**Date of HSCT** (2001-2010 vs 2011-2018)	0.7002	0.3186	1.5390	0.3750
**CR1/CR2**	3.5430	1.6420	7.6470	0.0013
***FLT3*-ITD/*CBF*r/*KMT2A*r**	1.5140	0.8187	2.7980	0.1862

SUPPLEMENTAL FIGURES

Figure S1

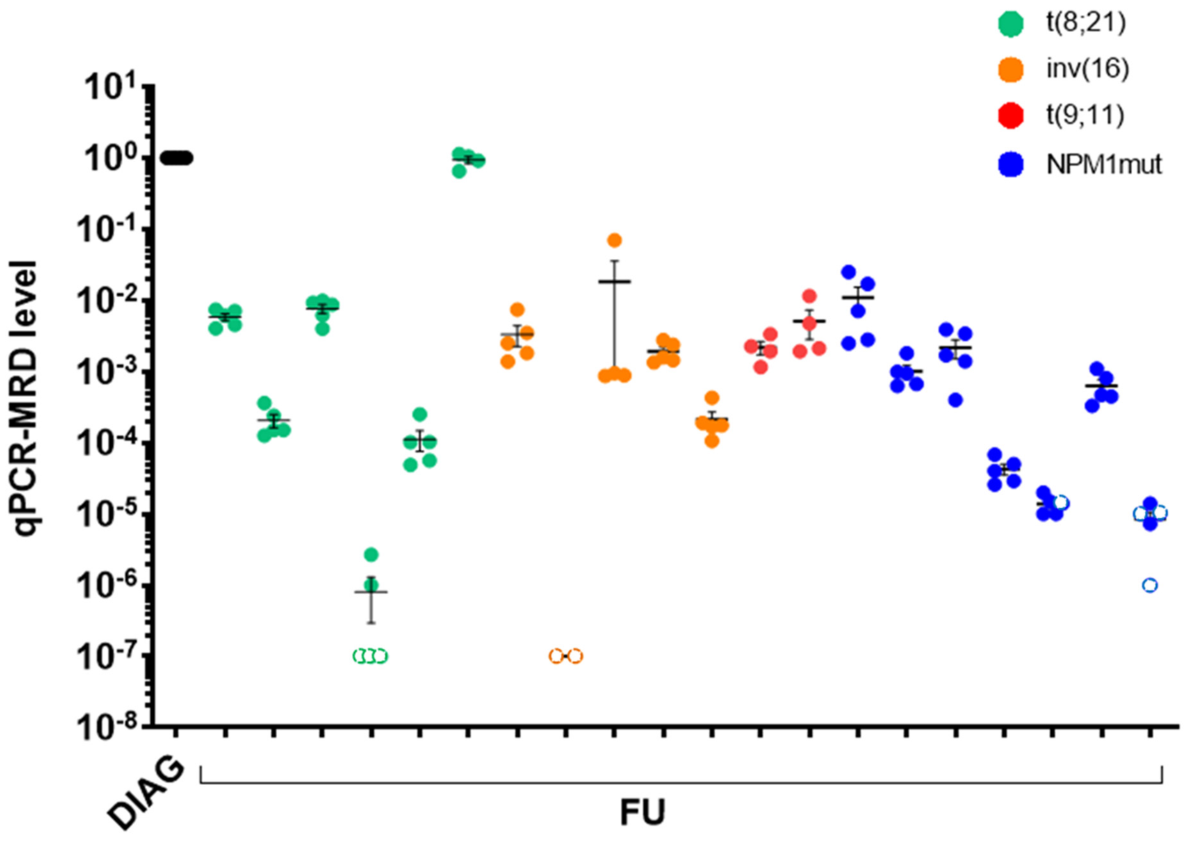

Figure S1. Inter-laboratory quality control (QC). Representative qPCR-MRD values plotted in the graph derived from 5 EU ref-laboratories in an inter-laboratory quality control pilot study. qPCR-MRD results collected for the same samples are concordant for all the molecular markers. DIAG, diagnosis; FU, follow-up samples. White circles with dotted line show undetectable levels of qPCR-MRD. Graph shows mean ± standard error mean.

Figure S2

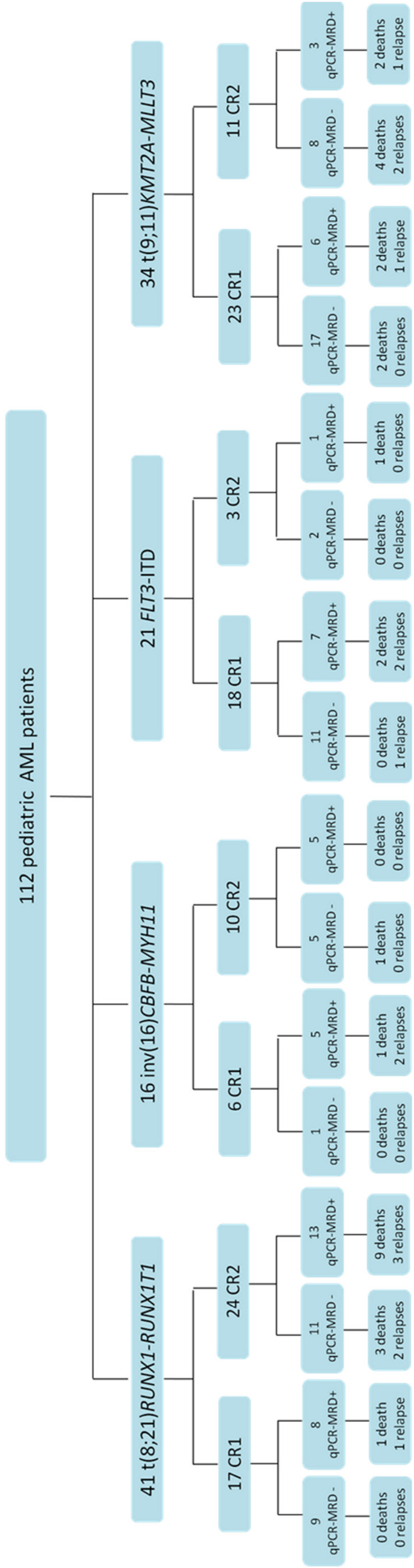

Figure S2. Schematic diagram showing the number of patients in each part of the study.

Figure S3

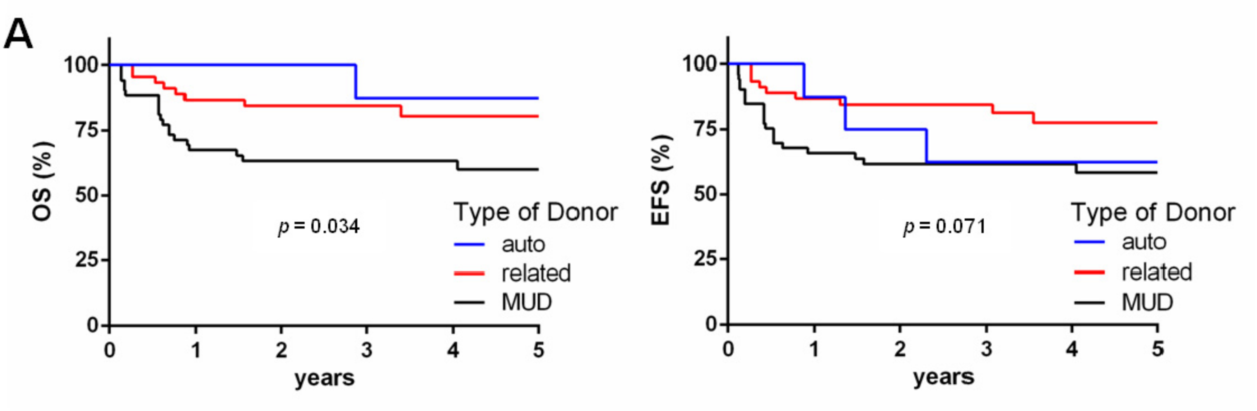

Number at risk:						
auto	8	8	8	6	4	4
related	47	38	34	28	16	15
MUD	53	32	28	24	20	17

Number at risk:						
auto	8	7	6	4	4	4
related	47	38	34	28	16	15
MUD	53	31	27	23	19	16

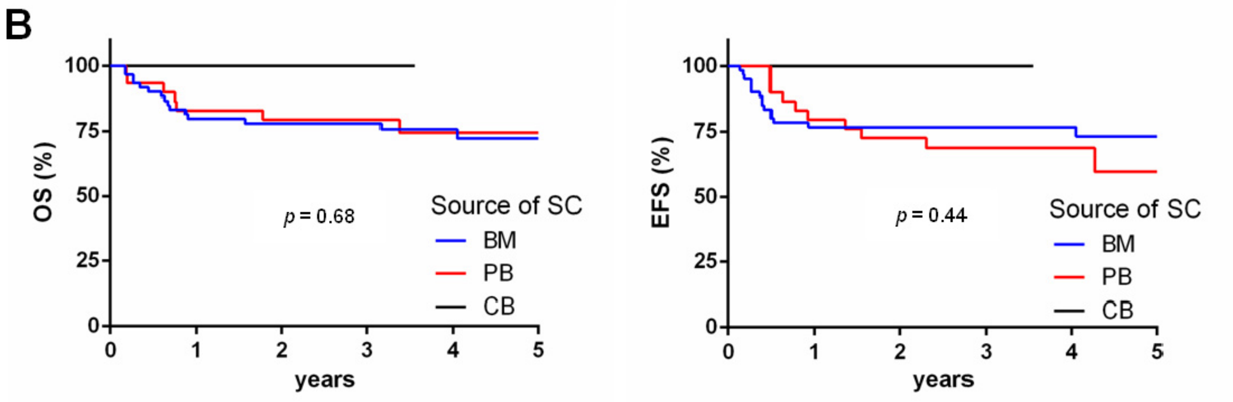

Number at risk:						
BM	61	44	39	32	22	21
PB	30	23	22	19	14	12
CB	3	2	2	2	0	0

Number at risk:						
BM	61	42	38	32	22	21
PB	30	23	20	15	13	11
CB	3	2	2	2	0	0

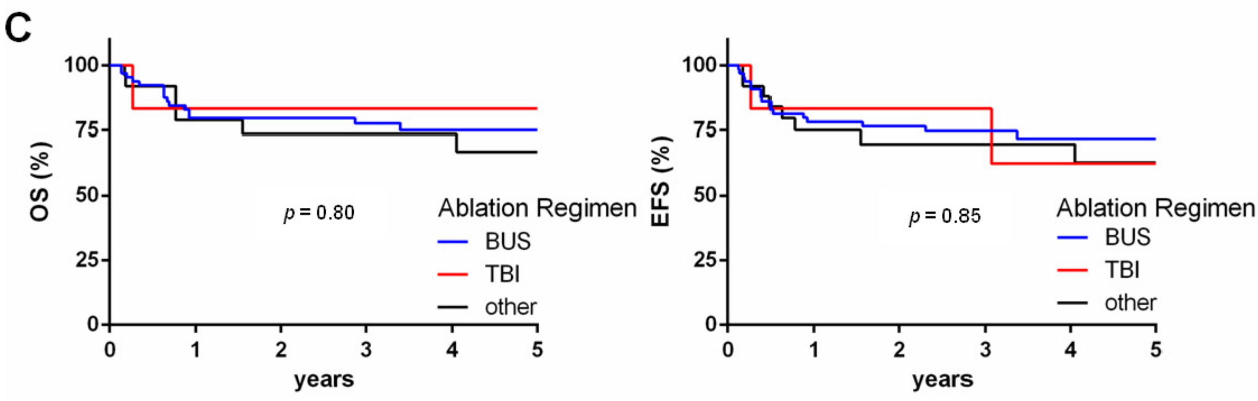

Number at risk:						
BUS	65	48	44	36	22	21
TBI	6	5	5	4	3	3
other	25	17	14	12	11	9

Number at risk:						
BUS	65	47	42	34	22	21
TBI	6	5	5	4	3	3
other	25	16	13	11	10	8

Figure S3. Effect of transplant-related variables on survival. Kaplan–Meier curves showing the effect of transplant-related variables in the entire cohort. (**A)** On the left, five-year OS for patients according to the type of donor: autologous (*n* = 8), related (*n* = 47) ,and MUD (matched unrelated donor, *n* = 53) (survival: 87.5% vs 80.5% vs 60.1%, *p* = 0.034). On the right, EFS (survival: 62.5% vs 77.7% vs 58.4%, *p* = 0.071); (**B)** OS (left) for patients according to the stem cell source used: bone marrow (BM) (*n* = 61), peripheral blood (PB) (*n* = 30), and cord blood (CB) (*n* = 3) (survival: 72.3% vs 74.5% vs 100%, *p* = 0.68); EFS is depicted on the right (survival: 73.3% vs 59.7% vs 100%, *p* = 0.44);(**C)** five-year survival curves comparing outcome of patients given busulfan-based (BUS, *n* = 65), total body irradiation (TBI) based (*n* = 6), or other (*n* = 25) ablation regimens. Probability of OS is 75% vs 83.3% vs 66.9% (*p* = 0.80); probability of EFS is 72% vs 62.5% vs 62.8% respectively (*p* = 0.85).

Figure S4

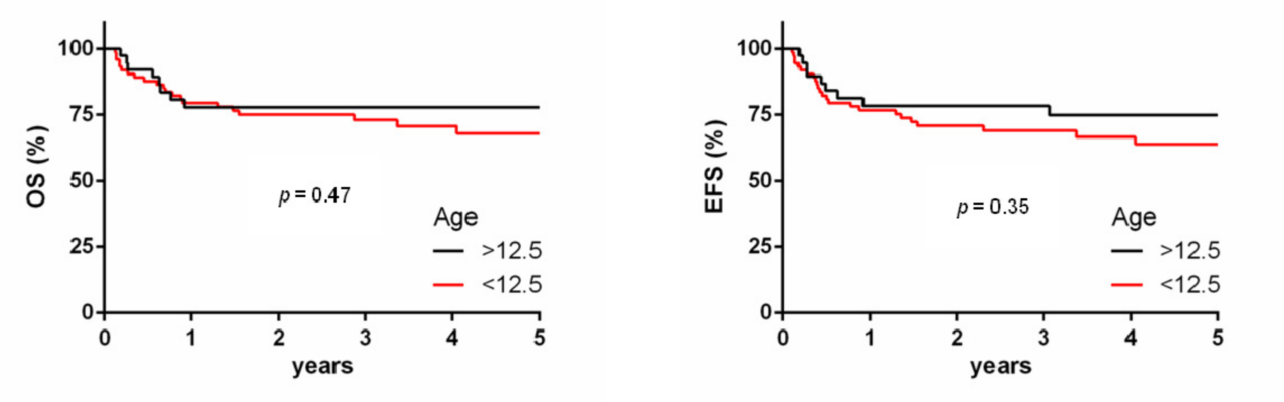

Number at risk:

>12.5	38	26	25	22	14	14
<12.5	74	55	46	36	26	22

Number at risk:

>12.5	38	26	25	22	14	14
<12.5	74	53	43	33	25	21

Figure S4. Impact of patient's age on clinical outcome. OS (left) for patients according to their age at diagnosis: survival for patients over 12.5 years of age (n = 38) was 77.8%, whereas for patients below 12.5 years (n = 74) it was 68% (p = 0.47). EFS is depicted on the right (survival: 74.8% vs 63.9% *vs* 100%, p = 0.35).

Figure S5

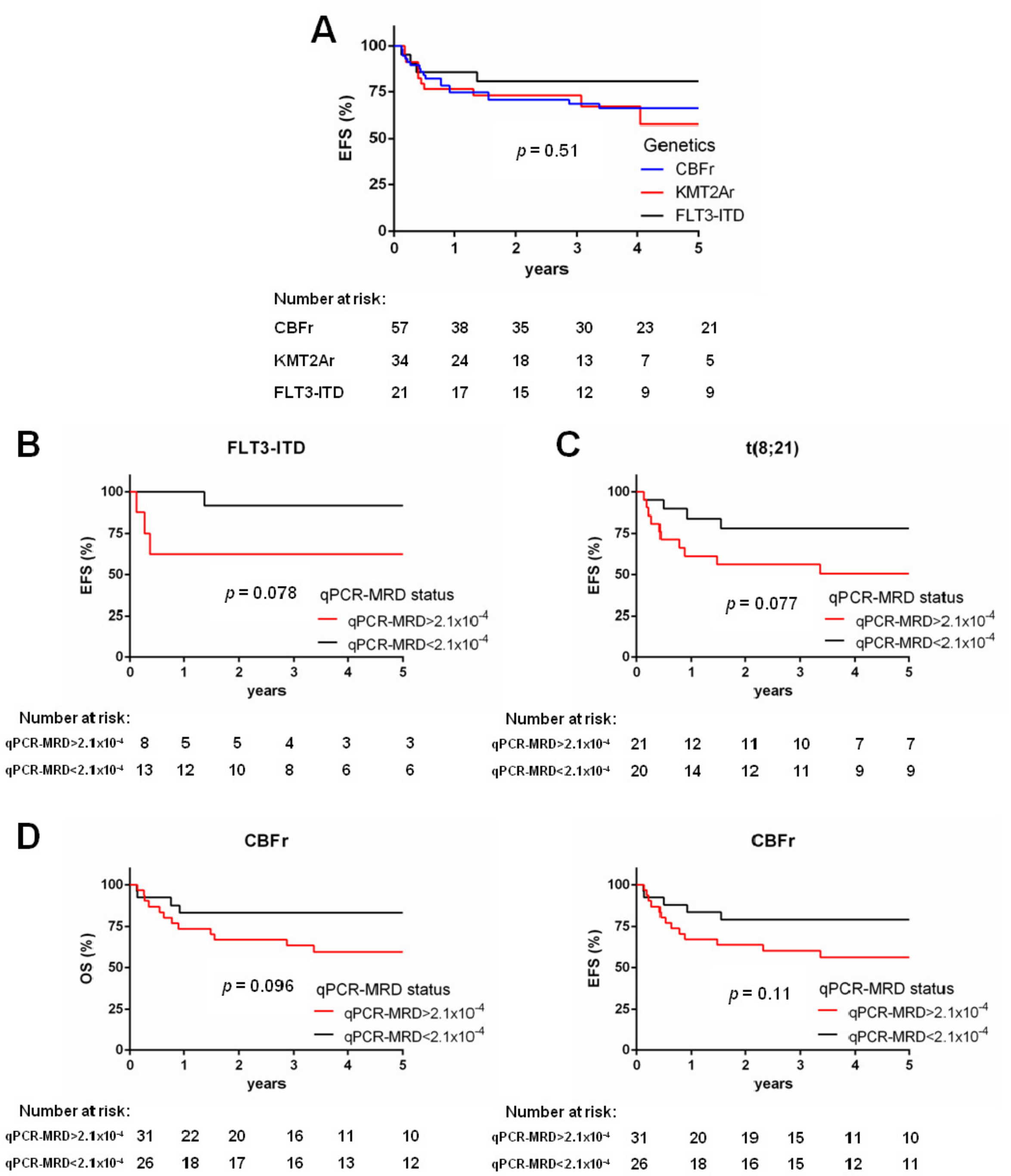
A
EFS (%)
100
75
50
25
0
0 1 2 3 4 5
years
p = 0.51
Genetics
CBFr
KMT2Ar
FLT3-ITD
Number at risk:
CBFr 57 38 35 30 23 21
KMT2Ar 34 24 18 13 7 5
FLT3-ITD 21 17 15 12 9 9
B
FLT3-ITD
EFS (%)
years
p = 0.078
qPCR-MRD status
qPCR-MRD>2.1x10-4
qPCR-MRD<2.1x10-4
Number at risk:
qPCR-MRD>2.1x10-4 8 5 5 4 3 3
qPCR-MRD<2.1x10-4 13 12 10 8 6 6
C
t(8;21)
EFS (%)
years
p = 0.077
qPCR-MRD status
qPCR-MRD>2.1x10-4
qPCR-MRD<2.1x10-4
Number at risk:
qPCR-MRD>2.1x10-4 21 12 11 10 7 7
qPCR-MRD<2.1x10-4 20 14 12 11 9 9
D
CBFr
OS (%)
years
p = 0.096
qPCR-MRD status
qPCR-MRD>2.1x10-4
qPCR-MRD<2.1x10-4
Number at risk:
qPCR-MRD>2.1x10-4 31 22 20 16 11 10
qPCR-MRD<2.1x10-4 26 18 17 16 13 12
CBFr
EFS (%)
years
p = 0.11
qPCR-MRD status
qPCR-MRD>2.1x10-4
qPCR-MRD<2.1x10-4
Number at risk:
qPCR-MRD>2.1x10-4 31 20 19 15 11 10
qPCR-MRD<2.1x10-4 26 18 16 15 12 11

E

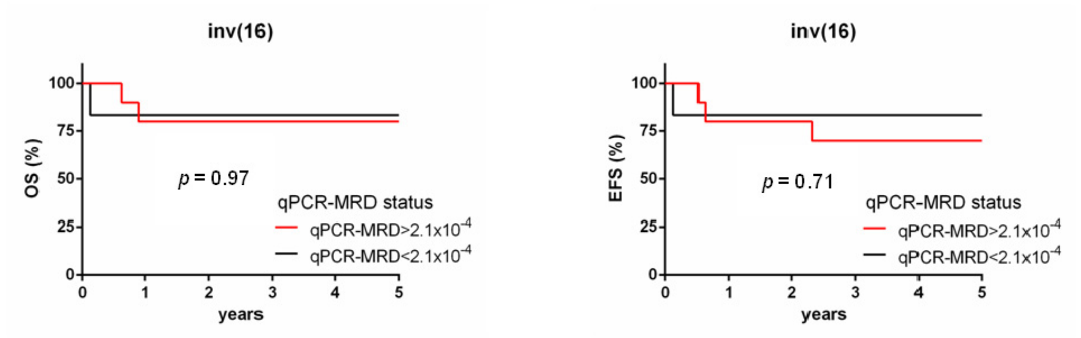

Number at risk:

qPCR-MRD$>2.1\times10^{-4}$	10	8	8	6	4	3
qPCR-MRD$<2.1\times10^{-4}$	6	4	4	4	3	2

Number at risk:

qPCR-MRD$>2.1\times10^{-4}$	10	8	8	5	4	3
qPCR-MRD$<2.1\times10^{-4}$	6	4	4	4	3	2

F

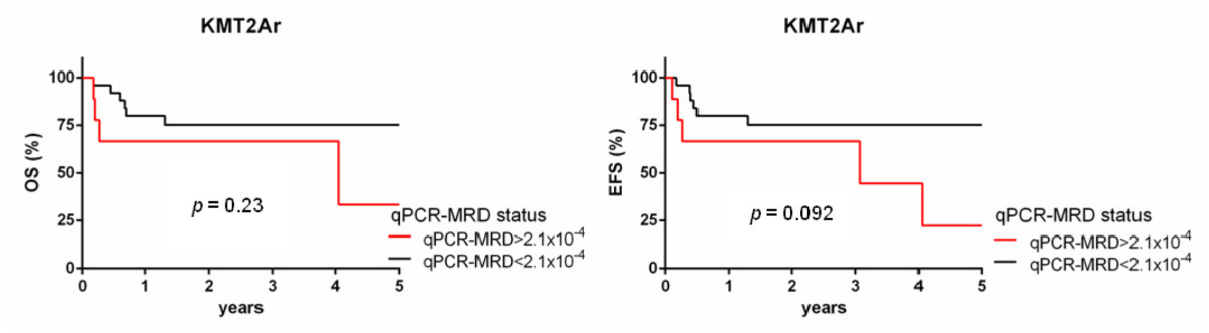

Number at risk:

qPCR-MRD$>2.1\times10^{-4}$	9	6	6	4	2	1
qPCR-MRD$<2.1\times10^{-4}$	25	18	12	9	5	4

Number at risk:

qPCR-MRD$>2.1\times10^{-4}$	9	6	6	4	2	1
qPCR-MRD$<2.1\times10^{-4}$	25	18	12	9	5	4

Figure S5. Genetics and qPCR-MRD status role in HSCT:(**A)** Five-year EFS of patients subdivided by genetic marker: *CBF*r (*n* = 57), *KMT2A*-rearranged (*n* = 34), and *FLT3*-ITD (*n* = 21) (survival: 66.1% vs 57.5% vs 80.7%, *p* = 0.51). (**B)** five-year probability of EFS for children with *FLT3*-ITD mutation given HSCT with qPCR-MRD value above 2.1 x 10^{-4} (*n* = 8) or with qPCR-MRD value below 2.1 x 10^{-4} (*n* = 13) (62.5% vs 91.7%, *p* = 0.078); (**C)** Kaplan–Meier survival curves of EFS for children with t(8;21)*RUNX1::RUNX1T1* translocation given transplantation with qPCR-MRD values above 2.1 x 10^{-4} (*n* = 21) or with qPCR-MRD values below 2.1 x 10^{-4} (*n* = 20) (50.5% vs 78.1%, *p* = 0.077); (**D)** five-year survival curves for patients with *CBF* rearrangements given HSCT with qPCR-MRD values above 2.1 x 10^{-4} (*n* = 31) or qPCR-MRD values below 2.1 x 10^{-4} (*n* = 26). On the left side, probability of OS (59.5% vs 83.5%, *p* = 0.096), on the right side, EFS (56.3% vs 79.2%, *p* = 0.11); (**E)** probability of OS (left) for children with inv(16)*CBFB::MYH11* given HSCT with qPCR-MRD values above 2.1 x 10^{-4} (*n* = 10, survival 80%) or qPCR-MRD values below 2.1 x 10^{-4} (*n* = 6, survival 83.3%) (*p* = 0.97) and, on the right, EFS (70% vs 83.3%, respectively, *p* = 0.71); (**F)** survival estimates for *KMT2A*-rearranged patients divided by qPCR-MRD values above 2.1x10^{-4} (*n* = 9) or below 2.1 x 10^{-4} (*n* = 25). OS (left) was 33.3% vs 75.3% (*p* = 0.23) and EFS (right) was 22.2% vs 75.3% (*p* = 0.092).

Figure S6

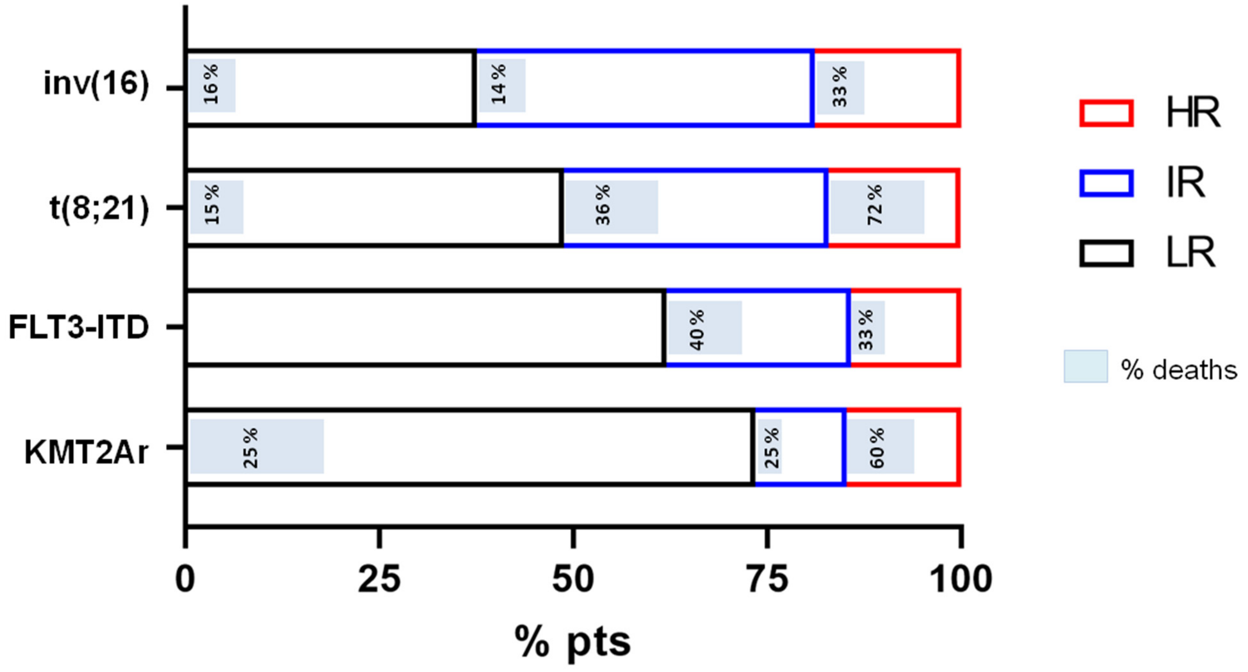

Figure S6. Bar representation of the percentage of patients belonging to LR, IR, and HR groups according to their genetic lesions. Grey bars represent the percentage of dead patients in each risk group.

Figure S7

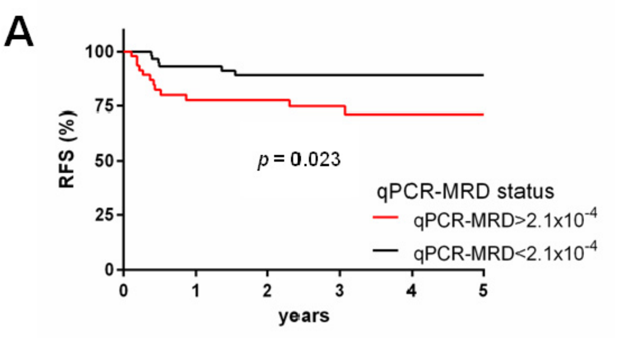

Number at risk:

qPCR-MRD>2.1x10-4	48	31	30	23	16	14
qPCR-MRD<2.1x10-4	64	48	38	32	23	21

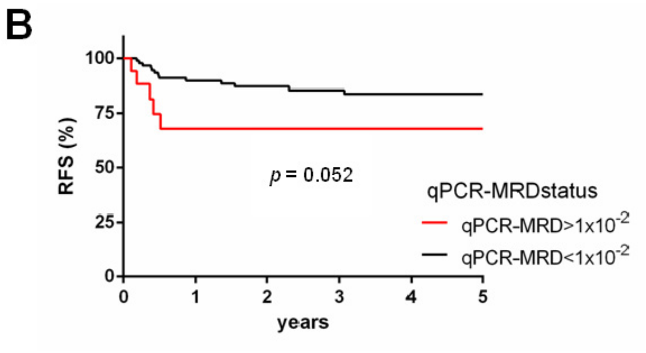

Number at risk:

qPCR-MRD>1x10-2	17	9	8	7	6	5
qPCR-MRD<1x10-2	95	70	60	48	33	30

Figure S7. Relapse-free survival from HSCT according to qPCR-MRD values. Relapse-free survival of patients according to (**A)** 2.1 x 10^{-4} cut-off (71.5% vs 89.2, p = 0.023); (**B)** 1 x 10^{-2} cut-off (67.9% vs 83.8%, p = 0.052).
